# Using Digital Tools to Advance Alzheimer's Drug Trials During a Pandemic: The EU/US CTAD Task Force

**DOI:** 10.14283/jpad.2021.36

**Published:** 2021-06-28

**Authors:** Jeffrey Kaye, P. Aisen, R. Amariglio, R. Au, C. Ballard, M. Carrillo, H. Fillit, T. Iwatsubo, G. Jimenez-Maggiora, S. Lovestone, F. Natanegara, K. Papp, M.E. Soto, M. Weiner, B. Vellas, the EU/US CTAD Task Force

**Affiliations:** 1 Layton Aging and Alzheimer's Disease Center, School of Medicine, Oregon Health and Science University, Portland, OR, USA; 2 Alzheimer's Therapeutic Research Institute, University of Southern California, San Diego, CA, USA; 3 Brigham and Women's Hospital, Harvard Medical School, Boston, MA, USA; 4 Boston University, Boston, MA, USA; 5 University of Exeter, Exeter, UK; 6 Alzheimer's Association, Chicago, IL, USA; 7 Alzheimer Drug Discovery Foundation, New York, NY, USA; 8 University of Tokyo, Tokyo, Japan; 9 Janssen Pharmaceutical Company, High Wycombe, UK; 10 Eli Lilly and Company, Indianapolis, IIN, USA; 11 Toulouse University Hospital, Toulouse, France; 12 University of California, San Francisco, CA, USA; 13 Gerontopole, INSERM U1027, Alzheimer's Disease Research and Clinical Center, Toulouse University Hospital, Toulouse, France

**Keywords:** Alzheimer's disease, clinical outcomes, digital tools, remote assessments

## Abstract

The 2020 COVID-19 pandemic has disrupted Alzheimer's disease (AD) clinical studies worldwide. Digital technologies may help minimize disruptions by enabling remote assessment of subtle cognitive and functional changes over the course of the disease. The EU/US Clinical Trials in Alzheimer's Disease (CTAD) Task Force met virtually in November 2020 to explore the opportunities and challenges associated with the use of digital technologies in AD clinical research. While recognizing the potential of digital tools to accelerate clinical trials, improve the engagement of diverse populations, capture clinically meaningful data, and lower costs, questions remain regarding the stability, validity, generalizability, and reproducibility of digital data. Substantial concerns also exist regarding regulatory acceptance and privacy. Nonetheless, the Task Force supported further exploration of digital technologies through collaboration and data sharing, noting the need for standardization of digital readouts. They also concluded that while it may be premature to employ remote assessments for trials of novel experimental medications, remote studies of non-invasive, multi-domain approaches may be feasible at this time.

## Introduction

The 2020 COVID-19 pandemic disrupted clinical trials worldwide as health care facilities became overwhelmed and lock-down conditions shuttered clinical trial sites, forcing both study staff and trial participants to stay home (1, 2, 3). Nearly every ongoing clinical study has felt the impact, although the degree of impact varies. Alzheimer's disease (AD) studies have been acutely affected for multiple reasons, including: 1) the increased vulnerability to COVID-19 of people with or at risk of AD due to their advanced age and high prevalence of comorbid conditions; 2) the effects of even mild cognitive decline on the ability of participants to comply with the operational changes in how trials are conducted (e.g., social distancing from study staff and study partners, changes in study protocols); and 3) effects on clinical endpoints due to isolation, confusion, and behavioral and psychological symptoms of dementia (BPSD) such as agitation, apathy, and depression (1, 4). Other disruptions common to all clinical trials including those for AD interventions include: 1) reduced clinical capacity; 2) increased costs due to the need for increased personal protective equipment (PPE) and infrastructure for altered intervention delivery; 3) delays in ethics approvals as institutional review boards (IRBs) are swamped with COVID-19 related protocols; 4) reluctance of patients and participants to be in contact with clinics and health providers; and 5) suspension of patient screening and recruitment.

Study sponsors have responded to these challenges in different ways; for example, by mailing drugs to trial participants to ensure participants have access to interventions; limiting clinic visits through the establishment of alternative trial sites; allowing a brief hiatus from dosing; and implementing home infusions. Funders have also responded with innovations. In an analysis of their funding portfolio during the latter half of 2020, the Alzheimer's Drug Discovery Foundation (ADDF) found that about 70% of grantees expected their studies to be delayed, and some requested no-cost extensions. Delays may harm not only budgets but data analyses as well, since data interpretation can be muddled due to long-term disruptions, missing data, and protocol deviations; with clinical studies more affected than preclinical studies. Observational studies have also been affected. For example, Swedish BioFINDER2 halted enrollment, and the Rush University aging cohort studies stopped performing autopsies due to lack of PPE. In response to these challenges, the Alzheimer's Association introduced a Rapid Program in Dementia (RAPID) funding program to provide bridge funding for Association-funded research during the pandemic.

In March 2020, the U.S. Food and Drug Administration (FDA) issued guidelines on the conduct of clinical trials during the pandemic and updated this guidance in December 2020 (5). The guidance aims to protect the safety of trial participants while ensuring good clinical practice and trial integrity. It also recognized the likely use of remote assessments and new technologies for data acquisition.

In recent years, digital technologies that enable remote assessment and gather real-world granular data on a variety of cognitive and functional outcomes have received increased attention and interest in the AD drug development community (6). Recognizing the urgency of developing these technologies due to the pandemic, the EU/US CTAD Task Force met in November 2020 to address the topic, bringing together an international group of clinical investigators from academia and industry along with funders. They explored emerging digital technologies that are being used in clinical research as well as the challenges that have yet to be addressed to realize the potential of these technologies. First and foremost, they recognized that global challenges such as the COVID-19 pandemic require global solutions.

## Opportunities and challenges to make trials more robust using digital technologies

Even if there were no pandemic, clinical trials as currently conducted are too slow and costly to adequately respond to a disease such as Alzheimer's, which currently affects around 50 million people worldwide and for which there is no approved disease-modifying treatment (7). The difficulty of conducting clinical trials for AD treatments is exacerbated by the use of conventional outcome measures that are insensitive to change early in disease and sensitive to environmentally-induced variation. By capturing alternative outcome measures in real-world settings, digital devices have the potential to mitigate these problems, thereby expediting trials, lowering costs, and enabling more effective clinical trials. Digital tools can also promote inclusiveness and social justice, increase the pool of participants, decrease sample size, and increase trial effectiveness by improving the generalizability and representation of vulnerable populations. Smartphones hold particular promise in the realm of digital tools since they are among the most penetrating technologies available, with over three billion users worldwide (8).

However, converting trial outcomes from in-person to remote measures is also associated with many challenges. Among the most critical aspects to be considered are usability and the reliability and fidelity of technology-related measures (9). Consumer-oriented devices and apps must be scaled for use in clinical trial settings and confounds introduced by remote assessment must be addressed. User training and support will be critical to reduce user errors. Technologies themselves should not drive their use in clinical trials; rather the use case should drive the technology.

In the AD space, digital tools have shown particular promise in capturing subtle cognitive and functional changes in preclinical AD. Yet there remain questions regarding their ability to serve as outcome measures due to questions around feasibility, reliability, and validity.

## Implementing digital devices in AD clinical studies

As the COVID-19 pandemic has necessitated moving clinical trials from specialized centers to home-based virtual assessments using both passive and active measures, investigators are learning lessons that have the potential to accelerate all clinical trials for dementia. Indirect measures of behavior, function, and physiology are being explored as cognitive and functional endpoints for clinical trials by individual research groups as well as technology companies. For example, Elektra Labs has created the Atlas platform, which takes a data science approach to clinical trials through the application of a wide selection of connected sensors and physiological and behavioral markers.

Many research groups that have developed and/or implemented digital technologies in clinical studies presented data and plans to the Task Force, providing lessons learned as well as strategies for moving forward:

### CART/ORCATECH Platform

**Figure 1:**
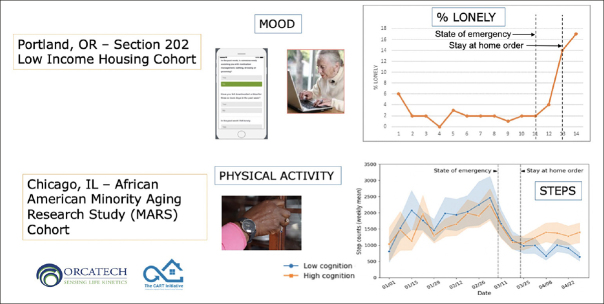
Pervasive Computing and Continuous Monitoring Demonstrate Effects of COVID-19 Pandemic on Loneliness and Physical Activity

The Collaborative Aging Research Using Technology (CART) Initiative funded by the US National Institutes of Health and the Department of Veterans Affairs, has developed a multi-functional digital assessment platform that is technology agnostic, use case flexible, sharable, and scalable (10). The platform, an extension of the Oregon Center for Aging & Technology (ORCATECH) assessment system at Oregon Health and Science University has been deployed to 301 older adults for up to three years in four US regions. It enables unobtrusive, long-term assessment across multiple domains (e.g., mobility, cognitive function, sleep) through the use of many different sensors and connected devices (e.g., electronic pillboxes, bed mats, actigraphs, online reporting on laptops, tablets or smartphones). The platform is currently deployed by multiple groups in the United States and Canada, with plans to extend the platform to France (in the community-wide INSPIRE study based at Toulouse University Hospital) and Australia. Testing in two cohorts — a low-income housing cohort in Portland, Oregon, and an African-American Minority Aging Research Study (MARS) cohort in Chicago — gathered data spanning the time period of the pandemic demonstrating increased loneliness and decreased physical activity (Figure 1). In a proof-of-concept trial, the platform has also demonstrated feasibility for monitoring and predicting agitation among residents of memory care facilities (11), suggesting that it may be possible to demonstrate the efficacy of treatments for agitation with sensors that monitor life-space activity.

In two separate cohorts, digital data streams provided by the CART ORCATECH platform demonstrated changes in mood (through online self-reported questionnaires) and physical activity (through actigraphy) that coincided with the imposition of stay-at-home orders because of the pandemic.

### RADAR-AD

The public private partnership Remote Assessment of Disease and Relapse — AD (RADAR-AD), funded by the Innovative Medicines Initiative (IMI) has embraced an “internet of things” (IOT) approach similar to the CART/ORCATECH system to measure early decline in cognitive function using low friction digital devices across a range of challenges. A study is planned that will enroll 200 participants over age 50 including those with preclinical AD, MCI due to AD, mild-to-moderate AD, and age and gender matched controls. RADAR earlier launched a multi-site prospective cohort study to determine the feasibility, usability, and acceptability of remote measurement technology (RMT) for major depressive disorder (MDD) (12).

Remote digital measurements that may be used in RADAR-AD include adaptations of multiple instruments to be administered using a table or smartphone interface such as the Amsterdam-instrumental activities of daily living questionnaire (A-IADL-Q) (13) to assess orientation, planning skills, and memory; and the Patient-Reported Outcomes Measurement Information Systems (PROMIS) general health measures to assess quality of life, disabilities, pain, and fatigue (14).

The RADAR base app, using a smartphone and passive sensors, will assess mobility, social communication, time at home, general activity, and sleep duration and quality. A residential movement detector will also assess gait and mobility at home and time spent in different areas of the home. The Mezurio smartphone app will capture high-frequency data related to cognition, including sleep, planning, memory, mood and anxiety, voice and speech, and typing and swiping (15).

### Harvard Aging Brain Study (HABS)

In its search for sensitive measures of subtle cognitive decline in preclinical AD, The Harvard Aging Brain Study (HABS) developed an iPad-based Computerized Cognitive Composite (C3) and subsequently a smartphone-based tool for remote assessment of memory. The C3 on the iPAD comprises 3 components: a Behavioral Pattern Separation task, a Face-Name Associative Memory Exam, and the Cogstate Brief Battery. Development of the C3 included its use in the A4 study, a secondary prevention trial in clinically normal older adults with abnormal levels of amyloid who were randomized to receive placebo or solanezumab. This initial study showed that when the drug was administered in screening period, the amyloid-positive group performed worse on C3 compared to the Preclinical Alzheimer Cognitive Composite (PACC) (16, 17). The C3 has also been shown to provide reliable data when participants complete the assessments at home on an iPad (18). The remote use of C3 allows for more frequent assessments, which provide new information about subtle cognitive changes (19).

HABS has also developed the Boston Remote Assessment for Neurocognitive Health (BRANCH), which combines two paired associative learning tasks, an associated memory test, and a continuous visual recognition task accessible on a smartphone. In a recent study, BRANCH was shown to be correlated with scores on the paper-based preclinical Alzheimer's cognitive composite (PACC5) (20).

HABS investigators have also been using BRANCH to assess learning curves using data from clinically normal older adults who completed 5 consecutive days of BRANCH. Early evidence suggests that a steeper learning curve is associated with better performance on the PACC, although the results were not statistically significant. Nonetheless, capturing learning curves using a smartphone over several days appears to be feasible and valid and may be useful to prescreen potential trial participants for biomarker status and identify those at greater risk for imminent cognitive decline and most likely to benefit from treatment. Cognitive learning curves may also serve as a paradigm for exploring psychopharmacokinetic profiles of medications to assess for efficacy over relatively short time periods.

### The Trial-Ready Cohort for Preclinical and Prodromal Alzheimer's Disease (TRC-PAD)

The Trial-Ready Cohort for Preclinical and Prodromal Alzheimer's Disease (TRC-PAD) was established to accelerate drug development for AD by ensuring timely recruitment of targeted individuals into optimally designed preclinical and prodromal trials. TRC-PAD gathers multiple types of data including digital data from wearables and sensors through its informatics platform (TRC-PAD IP). In concert with TRC-PAD, the Alzheimer's APT Webstudy registry was established along with an analytics platform, a referral management system, and a data management system (21).

The APT Webstudy has been working to optimize digital smartphone-based tools to assess cognition, such as the Cogstate Brief Battery. The study plans to implement this tool in the AHEAD 3–45 secondary prevention trials in early preclinical and preclinical AD and in the CT1812 trial in early AD (22). APT is also exploring alternative remote testing sensitive to amyloid elevation.

### WHO — Integrated Care for Older People (ICOPE) Monitor

In its World Report on Healthy Ageing, the World Health Organization (WHO) redefined healthy aging in terms of functional ability, which is a product of an individual's intrinsic capacity (IC) interacting with the environment (23). In order to implement integrated care for older people (ICOPE), an international partnership of researchers have developed a tool for assessing IC (24) as well as a mobile app available on smartphones that enable self-assessment by individuals to provide health care professionals with data on intrinsic capacity (25). Initial results from more than 5300 older individuals in the ICOPE Digital Cohort report high levels of IC impairments in multiple domains, including in vision, hearing, psychological health, and cognition. Recognizing that additional cognitive assessments are needed, ICOPE data will be combined with the Alzheimer's Prevention Trial (APT) Webstudy data beginning in 2021. The ICOPE integrative approach from WHO provides the opportunity to detect memory complaints or decline, taking into consideration declines in other ICs, such as vision, hearing, mood, nutrition.

### Brain Health Registry

With a goal of improving clinical trial recruitment, the Brain Health Registry (BHR) was established in 2014 as a website and online registry that collects digital data from potential clinical trial participants and study partners through self-report questionnaires and neuropsychological tests. Among the over 70,000 individuals enrolled in BHR, there have been over 106,000 referrals and 5,000 enrolled in other clinical studies. Through co-enrollment, BHR and clinical trial data can be linked.

BHR is also involved in methods development and validation of new online methods, including an electronic Clinical Dementia Rating (CDR) scale, a digital financial capacity instrument, and other digital assessment tools. In support of blood biomarkers studies, BHR has also collaborated with Quest Diagnostics to scale up blood collection using remote identification through the registry.

### Other Studies Incorporating Digital Assessment Tools

Many other clinical research programs are exploring using digital measures of cognition and function (e.g., web- or mobile- device based cognitive testing, automated speech and language assessment, or use of sensor-embedded home-based device responsives). For example, the Framingham Heart Study (FHS) is developing a smartphone platform with multiple applications including digital voice. Voice (spoken utterances and language) is measured through device agnostic technologies and FHS research shows that linguistic and acoustic measures can be used as biomarkers of cognitive function (26, 27, 28). However, while speech and language assessments are likely to be very sensitive, additional work is needed to determine the best way to capture speech samples.

The Deep and Frequent Phenotyping (DFP) study in the United Kingdom has also incorporated digital measures from wearables and smartphones to capture highly granular data on gait, memory, navigational ability, and other measures that may serve as proxies for cognition and function (29). The study aims to identify an optimal set of markers for patient stratification.

## Potential benefits and challenges associated with incorporating digital tools in trials

Digital devices offer the potential to expedite clinical trials, reduce the sample sizes or duration of studies, lower the cost of acquiring and analyzing data, enable the recruitment of more diverse and representative populations, reduce the need for specialized raters, reduce rater burden, and increase the clinical meaningfulness of outcome data collected to determine efficacy of an intervention. During a pandemic, digital tools also may provide the means for remote assessments, which could mitigate problems associated with closure of clinical trial sites and the safety concerns associated with in-person assessments and interventions.

Combining cognitive and functional endpoints in clinical trials is well established through the use of questionnaires in symptomatic stages of disease; and in preclinical disease, reports from both participants and study partners regarding high-level functional activities appear to provide valuable information on disease trajectory. Pairing digital cognitive measures with digital functional assessments may offer increased sensitivity in preclinical disease. The HABS team has shown that functional inventories can be administered remotely through the use of REDCap software, saving time and reducing data errors without sacrificing data quality.

The technologies employed in various digital devices provide additional potential advantages:

• They allow data to be time stamped, which enables assessment of everyday function.
• They can improve participant compliance by providing study participants and partners with reminders to take medications and engage in study-related activities.
• Audio and video recordings can provide a level of biometric validation to ensure that study participants themselves (rather than study partners) are providing data.
• Passive sensing of activities such as driving and computer use have demonstrated unique personalized patterns that can be captured using relatively simple algorithms.
• Passive sensors reduce participant burden, thus potentially reducing drop-outs and producing outcomes that are easily understandable and relevant in a real-world context.
• Meta-data such as time taken to complete a questionnaire or remember to take medication on time may also reflect changes in episodic memory, a key domain affected in AD.
• Passive assessment of sleep may prove useful since disrupted sleep has been shown to be a mediator of amyloid deposition in many studies.

To achieve the promise of digital devices will require addressing many challenges including those related to reliability, validation, user acceptability, regulatory approval, privacy and security. While regulators have indicated a willingness to consider digital data, much work remains to be completed before digital measures are accepted as primary or secondary outcomes. Indeed, the FDA issued guidance on the conduct of trials during a pandemic which includes increased flexibility around trial procedures intended to maintain participant safety, but less flexibility around efficacy measures. Task Force members also questioned whether regulators would accept remotely acquired data where there is no assurance that study participants and not study partners completed questionnaires or cognitive outcome assessments.

While digital registries may enable outreach to otherwise under-represented populations, engagement and retention of trial participants through digital means alone may be insufficient. Indeed, studies have reported high compliance rates that they attribute to personalized rather than anonymous digital engagement of study participants (30).

Transitioning cognitive assessments to smartphones may enable more frequent and sensitive measurement among large numbers of participants in a short period of time. However, smartphones may introduce additional challenges with regard to data fidelity and security. Other technological challenges related to digital data include the difficulty of analyzing continuous data and deconvoluting diverse data captured by devices. Addressing these challenges will be advanced by wider collaboration with the computer science and data science community.

Digital readouts are diverse and non-standardized, which results in impediments to data aggregation and sharing of data, both of which are essential to advance the field. Collaboration among technology developers is needed to ensure that raw data from which readouts on different devices are derived will be made available to the research community. Privacy concerns represent an additional roadblock to data sharing. Federated data management platforms have been developed that provide researchers with access to multiple types of data from multiple sources (31). The Alzheimer's Disease Data Initiative has also established the AD Workbench (ADWB) platform to promote data, information, and tool sharing across the AD research community (32), although no digital biomarker data are yet incorporated in this relatively new effort.

## Moving Forward

Despite the challenges of conducting clinical studies during the pandemic, the AD clinical research community remains committed to advancing current studies and launching new studies. Indeed, Task Force participants suggested that the COVID-19 experience may fuel preparations for better clinical trials that mitigate both the risks associated with the pandemic and more common complications of clinical trials for example by broadening the reach of recruitment efforts.

Among the possibilities discussed by the Task Force was the potential to design completely digital or hybrid trials combining virtual/telemedicine and physical visits. For example, telemedicine consultations in the pre-screening phase of a trial could help verify inclusion and exclusion criteria, provide information to potential trial participants, and obtain electronic consent. Virtual visits could also be used for trial components that do not require technically invasive procedures. The INSPIRE study team at Toulouse University Hospital tested this strategy following the first COVID lockdown in France and found a high rate of acceptance among participants (33).

Digital devices may provide new analytical methods as well, for example, by enabling assessment of intraindividual change in a high frequency way. Digital tools implemented during the pandemic may also provide increased understanding to the effects of daily stress, anxiety, and poor sleep on cognitive outcomes. They may also enable continued assessment of cognitive function in participants post-infection with COVID-19.

To ensure that clinical studies continue through the pandemic and its aftermath, the Task Force recommended that regulators and institutional review boards provide guidance yet maintain flexibility with regard to changes in applications/devices and statistical plans and allow for virtual and home-based visits and remote monitoring. Developers must consider alternate or additional trial sites, and funders should expect unanticipated modifications to protocols and funding needs.

The Task Force concluded its session with a discussion of the field's readiness to conduct remote clinical trials. Task Force members urged caution in employing remote assessments for trials of experimental medications; the digital measures at this time may be most productively embedded in parallel with conventional measures in early-stage Phase 1 and 2 trials, demonstrating their fidelity or even superiority to current commonly used clinical outcome measures. Enthusiasm was expressed for using digital technologies in remote studies of non-invasive interventions such as studies of vitamins, exercise, and mind-body or multidomain approaches. Trials of repurposed drugs for which safety data exists may also be feasible as entirely remote trials. For example, because of the COVID-19 pandemic, the Alzheimer's Disease Cooperative Study (ADCS) is attempting to conduct the PEACE-AD (prazosin for disruptive agitation in Alzheimer's disease) trial (34) as a remote-only trial.
